# Cardiac cephalalgia: one case with cortical hypoperfusion in headaches and literature review

**DOI:** 10.1186/s10194-017-0732-3

**Published:** 2017-02-20

**Authors:** Miao Wang, Lu Wang, Changfu Liu, Xiangbing Bian, Zhao Dong, Shengyuan Yu

**Affiliations:** 1 The Department of Geriatric Neurology, Chinese PLA General Hospital, Beijing, China; 2 The Outpatient Department of Fuxing Road No. 7, the First Affiliated Hospital of PLA General Hospital, Beijing, China; 3 The Department of of Cardiology, Chinese PLA General Hospital, Beijing, China; 4 The Department of of Radiology, Chinese PLA General Hospital, Beijing, China; 5 Department of Neurology, Chinese PLA General Hospital, Fuxing Road 28, Haidian District, Beijing, 100853 China

**Keywords:** Cardiac cephalalgia, Clinical features, Neuroimages, Pathophysiology

## Abstract

**Background:**

Cardiac cephalalgia (CC) is a rare disease occurring during an episode of myocardial ischemia and relieved by nitroglycerine. Though more than 30 cases of CC have been reported since 1997, the mechanism is yet obscure. Herein, a case of CC is presented and discussed in relevance with previous literature to propose a novel hypothesis about the mechanism of CC.

**Method:**

A CC patient with cortical hypoperfusion during headache attacks was presented, which has never been reported. All published cases of CC via PubMed (http://www.ncbi.nlm.nih.gov/pubmed) in English literature, between 1997 and 2016, were reviewed.

**Results:**

A patient suffering from CC presented a cerebral hypoperfusion during a headache attack. This phenomenon had not been observed since CC was introduced in 1997. The literature review summarized the clinical presentations, neuroimaging features, ECG, and coronary angiography features of 35 CC patients.

**Conclusion:**

Based on the phenomenon of hypoperfusion in the event of a headache, the vessel constriction hypothesis was proposed including two potential physiological mechanisms underlying the pathophysiology of CC.

## Introduction

Headaches associated with exertion or sexual activities have been regarded as benign if structural lesions can be excluded. In 1997, Lipton et al. summarized two current and five previous cases of an exertional headache complicated with acute coronary syndrome and discovered that the headache was relieved by treatments for acute coronary syndrome, such as the administration of nitroglycerine and/or surgical interventions including coronary artery bypass grafting or percutaneous angioplasty [1]. Thus, they deemed it a rare type of an exertional headache and suggested the term “cardiac cephalalgia” (CC) describing the type of headache, which may have life-threatening implications if misdiagnosed. Since 1997, more than 20 reports of CC have been reported; however, the pathogenesis remains unclear. Hitherto, three hypotheses were proposed to illustrate the mechanism of CC: convergence of nerve fibers within the spinal cord, increased intracranial pressure secondary to decreased venous return from the brain, and increased inflammatory mediators causing vasodilation. The present paper aims to delineate the clinical features of CC and put forth a prospective mechanism.

## Materials and methods

### Case and literature review

We described the clinical features as well as the neuroimaging data of CC patients, and searched PubMed database using the terms “cardiac cephalalgia”, “cardiac cephalgia”, “headache and angina”, “headache and acute coronary syndrome”, and “headache and myocardial infarction”. The following limitations were exercised: full text, English language only, and published after 1997.

## Results

### Case presentation

A 40-year-old male presented a 4-year history of episodic bitemporal headaches before he was seen for neurological consultation in outpatient. The headaches were rated as 7–10 in severity on the visual analog scale, pulsatile, tight in quality, and occasionally radiating to upper limbs. The headaches were sometimes also associated with chest discomfort, palpitations, cold sweating, and facial pallor. However, the patient denied nausea, vomiting, photophobia, and phonophobia.

The symptoms attack occurred 2–3 times per month, elicited by exertion, cold stimuli, and sexual activities, lasting 5–10 min, and relieved after treatment with nitrates. Coughing, sneezing, or having a bowel movement did not trigger the pain.

The patient self-administered aspirins and statins post-diagnosis of acute non-ST-elevation myocardial infarction (NSTEMI) in 2009 and nifedipine to control hypertension since 2001. Additionally, he presented 20 years of smoking history with 30 cigarettes/day but has ceased smoking for 5 years before the start of headache.

**Fig. 1:**
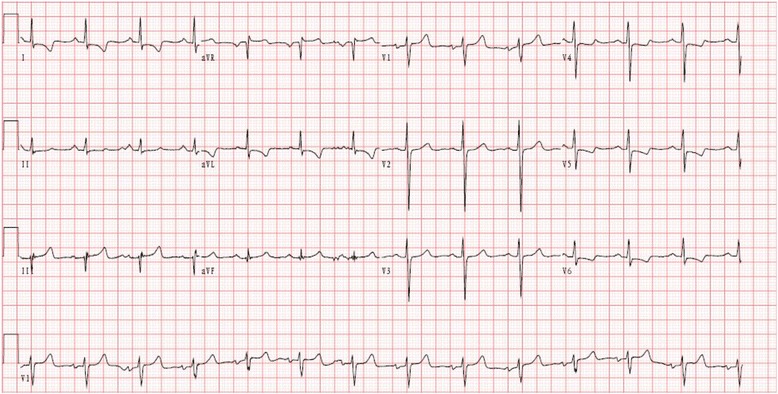
The ECG showed inverted T wave

Physical examination revealed normal blood pressure, heart rate and rhythm, systemic and neurological examination results. The cardiac enzymes were in normal range at the time of headache attack. The estimation of catecholamines and their metabolites were normal. The ECG showed inverted T wave (Fig. 1).

The patient underwent brain MR examinations with routine clinical sequences including axial T1W, T2 FLAIR, diffusion-weighted imaging (DWI), and MRA (Magnetic Resonance Angiography) on a 3.0 T MR system (Discovery MR750, GE Healthcare, Milwaukee, WI, USA) equipped with an 8-channel head coil to receive signals. Perfusion-weighted images (PWI) were obtained using a 3D pCASL technique.

**Fig. 2:**
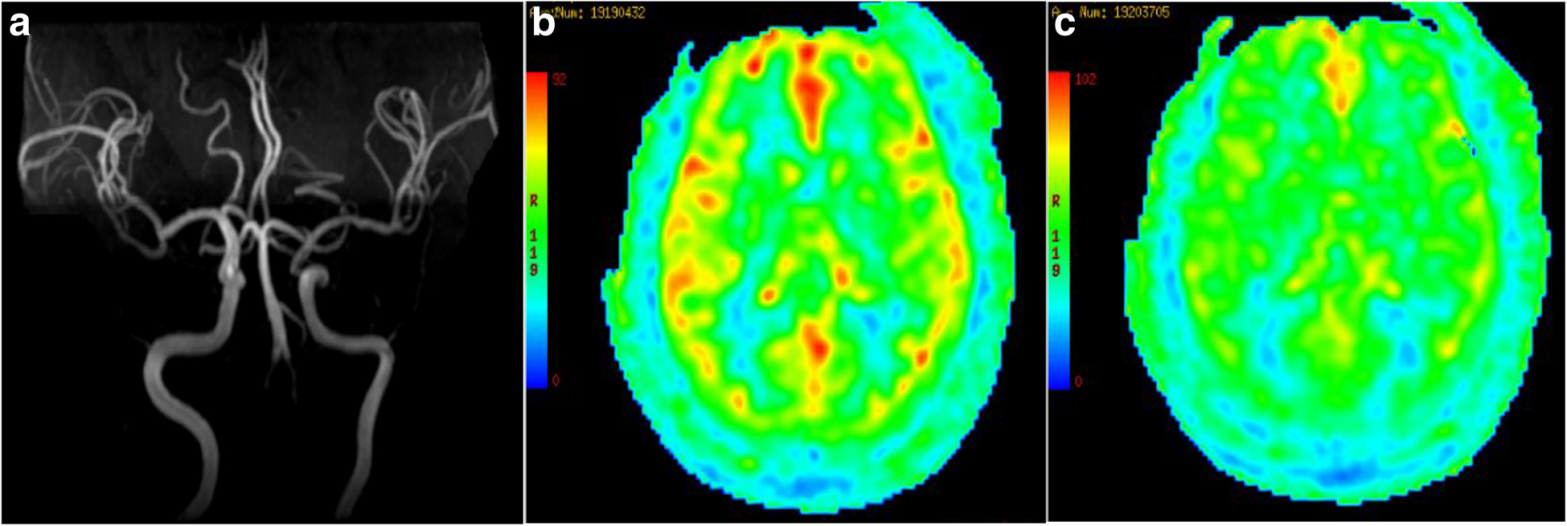
MRA (**a**) was negative when headache attack. PWI obtained during the headache not attack (**b**). PWI obtained during headache attack (**c**)

T1, T2, FLAIR and DWI weighted images of brain MRI were negative (Fig. 2a). However, the PWI revealed cerebral hypoperfusion during the headache attacks (Fig. 2b, c).

**Fig. 3:**
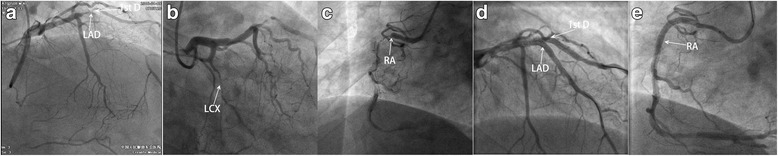
Coronary angiography demonstrated complete occlusion at the middle segment of *left* anterior descending (LAD) and proximal *right* coronary arteries (RCA), 80% stenosis at the *middle* segment of left circumflex (LCX), and 80% stenosis at the bifurcation of the first diagonal (**a**, **b**, **c**). Percutaneous transluminal angiography (PTCA), stenting of LAD, and bifurcation of the first diagonal and the RCA were carried out successfully (**d**, **e**)

Owing to the headaches provoked by exertion, cold stimuli, and sexual activities and relieved after administration of nitrates, CC diagnosis should be suspected according to the international classification of headache disorder (ICHD-3β) in 2013 [2]. Thus, a coronary angiography was performed, which demonstrated complete occlusion at the middle segment of left anterior descending (LAD) and proximal right coronary arteries (RCA), 80% stenosis at the middle segment of left circumflex (LCX), and 80% stenosis at the bifurcation of the first diagonal (Fig. 3). Percutaneous transluminal coronary angiography (PTCA), stenting of LAD, and bifurcation of the first diagonal and the RCA were carried out successfully. Six months following the operation, the patient did not report any recurrence of a headache.

### Literature review

The demographic data and the clinical manifestations of the 35 cases were indicated in Tables 1 and 2. A male predominance was observed (male:female ratio 1.5:1). The mean age was 62-years-old.

**Table 1:** Clinical manifestations of cardiac cephalalgia headache

A. Sexual characteristic	
M/F	21/14
B. Triggers	
Expertise/Sexual/Emotion	18
None	6
Not mentioned	11
C. Side of headache	
Right	3
Left	2
Bilateral	16
Not mentioned	14
D. Regions of headache	
Occipital	16
Vertex	6
Frontal	7
Temporal	5
Parietal	3
Other Regions^a^	
E. Quality of headache	
Shooting	4
Bursting	4
Sharp	5
Dull	6
Squeezing	2
Pulsating	2
Not mentioned	16
F. Intensity of headache	
33 cases presented severe intensity. 2 cases presented mild or moderate	
G. Associations with headache	
Nausea or Vomiting	10
Sonophobia	1
Photophobia	1
Sweating	5
Dizziness	2
Other Associations^b^	
Without	21

Other Regions^a^:2 cases radiate to shoulders. 2 case presented full head pain. 1case presented right eyeball pain. 1 case had not mentioned

Other Associations^b^:1 case associated with Confusion, agitation

**Table 2:** The clinical presentations of Cardiac ischemia

Source	Sex/Age	Risk Factors	Symptoms of Cardiac ischemia	Cardiac Enzyme	ECG	Stress Test	DSA
Grace, 1997 [1]	M/59	None	None	NA	T inversion in anterolateral leads	ST depression	RCA occlusion, LAD 70% stenosis
Lipton, 1997 [31]	M/57	Smoking	Vague abdominal, chest discomfort	NA	Flattened T in V2, AVL. Inverted T in V5. Biphasic T waves in V3, V4, V6	ST depression in II, III, aVF and V4-6	Severe three-vessel disease
	M/67	Hypertension, Smoking	None	NA	Sinus bradycardia and a possible old inferior wall myocardial infarct	ST depression in inferolateral leads	Three-vessel disease with 90% LCX stenosis
Lance, 1998 [32]	M/62	Hyperlipidemia, Smoking	Tightness in the right side chest	NA	NA	ST depression in V4-V6	LAD and RCA occlusion
Lanza, 2000 [3]	M/65	Hyperlipidemia, Smoking	None	Elevate	Normal	T peaking in V2-V4 leads	RCA occlusion, LAD and LCX 90% stenosis
Amendo, 2001 [6]	F/78	Hyperlipidemia, Hypertension	None	Elevate	ST elevation in I, aVL	NA	Severe triple vessel disease
	F/77	None	None	Elevate	T inversion across the precordium	NA	Normal coronary arteries
Rambihar, 2001 [33]	F/56	None	None	NA	NA	ST depression in I, II, AVF, V4-6 leads	LM 40% stenosis, 90% LCX 90%, stenosis, LAD 99% stenosis
Auer, 2001 [34]	M/47	Hyperlipidemia, Smoking	NA	NA	ST elevation in I, aVL; ST depression in II, III, aVF	NA	Biopsy: LAD occlusion, RCA 80% stenosis
Martínez, 2002 [35]	F/68	Hyperlipidemia, Diabetes, Smoking	None	NA	T inversion in V2, V3, and V4.	ST depression in anterior, lateral, and septal	Three-vessel occlusive disease.
Famularo, 2002 [36]	M/70	Hypertension, Smoking	Midepigastric pain	Elevate	ST elevation in II. III, aVF	NA	Not performed
Morlote, 2002 [37]	M/59	Hyperlipidemia, Hypertension	Chest discomfort, diaphoresis.	NA	ST depression	NA	NA
Source	Sex/Age	Risk Factors	Symptoms of Cardiac ischemia	Cardiac Enzyme	ECG	Stress Test	DSA
Sathirapanya, 2004 [38]	M/58	Smoking	Chest tightness	NA	ST elevation in V1-3	NA	LM 50% stenosis, LAD occlusion, 1st OM 70% stenosis, RCX occlusion
Chen, 2004 [39]	M/76	Hypertension	Chest pain	NA	T inversion in I, aVL	ST depression in II, III, aVF	LAD 90% stenosis, RCA 50% stenosis
Korantzopoulos, 2005 [40]	F/73	Hyperlipidemia, Hypertension, Obesity	None	Elevate	ST depression in V2-V5	NA	LAD 70% stenosis
Cutrer, 2006 [5]	M/53	Hyperlipidemia, Hypertension, Smoking, Obesity	None	NA	NA	Negative	RCA occlusion, LAD 60% stenosis
Morlote, 2006 [41]	F/74	Diabetes, Obesity	Chest tightness	Evaluate	ST depression in V1-V4	NA	Not performed
	F/64	Hyperlipidemia, Hypertension, Diabetes	None	Not performed	Not performed	Not performed	Not performed
Seow, 2007 [42]	M/35	Smoking	None	Evaluate	ST evaluate in V2 to V4	NA	LAD occlusion
Broner, 2007 [43]	F/72	Hyperlipidemia, Diabetes	None	Evaluate	ST evaluate in II, III, aVF	NA	RCA occlusion, LAD 30% stenosis
Wei, 2008 [28]	M/36	Smoking	Ventricular fibrillation with cardiac arrest	NA	ST evaluate in V2 to V6	NA	LAD 90% stenosis
	F/85	None	Chest pain	NA	NA	NA	NA
Wang, 2008 [29]	F/81	Hypertension	Loss consciousness, ventricular fibrillation.	NA	ST evaluate in II, III, aVF	NA	RCX 99% stenosis
Source	Sex/Age		Symptoms of Cardiac ischemia	Cardiac Enzyme	ECG	Stress Test	DSA
Dalzell, 2009 [44]	F/44	Smoking	None	NA	ST evaluate in II, III, aVF	NA	RCA occlusion
Sendovski, 2009 [45]	F/65	Hyperlipidemia, Hypertension,	None	Evaluate	ST depression at lateral wall and a mild ST elevation at precordial leads.	NA	LAD 70% stenosis, LCX 95% stenosis, RCA 80% stenosis, RPD 90% stenosis
Chatzizisis, 2010 [46]	M/42	Diabetes	None	Evaluate	ST depression and invert T in V2-V5	NA	LAD occlusion
Yang, 2010 [4]	F/44	None	Chest discomfort, epigastric pain	NA	Normal	ST depression	Vasospasm of the right coronary artery in response to an intra-arterial injection of acetylcholine
Costopoulos, 2011 [47]	M/55	Hyperlipidemia, Hypertension, Smoking, Diabetes	Breathlessness	Evaluate	Widespread ST segment depression	NA	Triple-vessel disease
Elgharably, 2013 [48]	M/55	Smoking	None	Evaluate	Q wave in III, aVF	NA	Significant lesion with thrombus in LAD
Asvestas, 2014 [49]	M/86	Hypertension, Smoking	None	Evaluate	ST depression in V1-V5, ST elevation in V7-V9	NA	LCX occlusion, LAD and RCA severe stenosis
Mathew, 2014 [50]	M/42	Hypertension	Palpitations, shortness of breath	NA	NA	NA	LAD 99% stenosis
Prakash, 2015 [51]	M/67	Smoking	NA	NA	Q in II, III, aVF.	ST depression in inferior leads.	RCA 90% stenosis, LAD 75% stenosis, LCX 70% stenosis
Huang, 2016 [52]	F/70	Hypertension, Smoking	None	Evaluate	ST elevation in V2-V6	NA	Significant stenosis with intramural thrombus of LAD.
Shankar, 2016 [53]	M/73	Diabetes	Chest pain	NA	Normal	ST depression in inferior leads.	Triple-vessel disease
Current case	M/40	Smoking	Chest discomfort, palpitations	Normal	Inverted T wave	Not performed	Triple-vessel disease

The clinical manifestations of the headache were displayed in Table 1. In more than half of the patients, the headache could be trigged in conditions with high myocardial oxygen consumption, such as exertion, sexual activity, and emotional fluctuation. However, 6 cases have been reported; wherein the headache appeared during rest. The symptom was not localized in a specific area but involved frontal, temporal, parietal, and occipital regions. Moreover, the headache may be unilateral or bilateral. The quality of a headache was varied including shooting, bursting, dull, and squeezing. A maximum number of patients had a severe intensity of headache, and only two patients presented mild or moderate. In some cases, the headache had no accompanying symptoms while in others, it was accompanied by photophobia, phonophobia, osmophobia, and nausea or vomiting. Notably, all the reviewed cases of CC described the resolution of a headache after reinstating the flow in cardiac vessels by medical or surgical interventions.

The clinical manifestations of cardiac ischemia were illustrated in Table 2. About 1/2 of the CC patients (18 cases) were without typical angina symptoms, which included chest pain or tightness, palpitations, and dyspnea. Most of the patients showed pathological alterations of the baseline ECG trace, such as ST-segment elevations or depressions and T-wave inversions, as well as, elevated cardiac enzymes. However, three separate cases presented normal ECG at rest [3, 4], and one case presented a negative ECG even under stress [5]. Thirty-one out of 35 cases underwent coronary artery stenosis or occlusion. However, normal coronary angiography results could not exclude the diagnosis of CC as two cases continued to present normal coronary angiography [4, 6]. After intra-arterial injection of acetylcholine in 1 of the cases, coronary artery’s contraction was observed. These two cases were suggested as potential variants of angina.

## Discussion

### Clinical features

In “typical” cases that were triggered by exertion or sexual activity or emotion fluctuation and accompanied by the symptoms of angina, a diagnosis of CC could be established according to the medical history demonstrating the exact onset of headache concurrently with acute myocardial ischemia, abnormality of ECGs performed at rest or under stress, elevating cardiac markers (CPK-MB, myoglobin, and troponin) and coronary angiography illustrating coronary arteries occlusion or stenosis. However, the majority of cases were “atypical”. Headaches may occur as the sole symptom without the symptoms of angina, absence of triggers, absence of ECG abnormalities (Table 2) when acute myocardial ischemia onset. In “atypical” cases, the doctors might be prone to omit the cardiac examinations (cardiac marker and coronary angiography) and make an incorrect diagnosis of benign headaches related to exertion, cough, migraine, and even orgasm. Especially, when some cases resembled migraine without aura, we should be able to make a diagnosis and prescribe triptans, which are vasoconstrictors aggravating the cardiac ischemic. However, there were more clinical clues implying the diagnosis of CC. We observed that most of the patients were above 50 years of age, and the majority of them presented cardiac risk factors such as hypertension, hyperlipidemia, diabetes, and smoking. Therefore, it is suggested that the patients suffering a new headache, who are over 50-years-old or display cardiac risk factors, should be suspected of CC. We also found that the headaches of all the patients reacted to vasodilators. Also, we demonstrated that the experimental treatment by vasodilators might be an efficient method in the event of difficulty in differentiating CC from other headaches. Moreover, even the patient showed a normal ECG; thus, the diagnosis of CC should not be excluded, because CC patients could have a normal ECG even under the stress test [3, 4]. Therefore, we suggested that coronary angiography should be recommended to all patients with suspected CC for accurate diagnosis.

### Pathophysiology

Since the diagnosis had been introduced in 1997, several theories have been proposed about the pathogenesis of CC. Based on the current case, we proposed a new hypothesis about the mechanisms of CC vessel constriction hypothesis.

In the current case, the PWI of brain MRI confirmed the cerebral hypoperfusion; however, MRA was normal during the headache attack, which had not been reported. Thus, this phenomenon might lead to headache by two possible physiological mechanisms. Firstly, the hypoperfusion may suggest reversible microvessels constriction during headache. The reversible cerebral vasoconstriction syndrome (RCVS) has been classified with moderate to a severe headache, which was also accompanied by reversible vasoconstriction of the cerebral vasculature [2]. Since the reversible vessels constriction could be restored both in CC and RCVS, we speculated that they might share some common mechanisms. Several previous studies revealed that vasoconstriction of RCVS was associated with sympathetic over-activity based on clinical observations or hypotheses including central vascular tone changes, aberrant sympathetic response, pheochromocytoma, and autonomic dysreflexia [7–10]. The mechanism of CC might also be linked to sympathetic hyperfunction due to the following reason: when myocardial ischemia occurred, the cardiac sympathetic afferents nerve could be stimulated. Hence, the activation of sympathetic afferents nerve could increase the sympathetic outflow through cardiac sympathetic nerve reflexes, which has been confirmed by several physiological tests [11–13].

On the other hand, sympathetic hyperfunction and parasympathetic hypofunction were found in migraine patients [14, 15]. And the autonomic nervous system imbalance might be derived from the abnormal functional connections between the hypothalamus and other brain structures involved in autonomic function including the brain stem [16]. Thus, we speculated that the sympathetic hyperfunction in CC patients might also be associated with abnormal hypothalamic functional connectivity. Although the distinguishing characteristics of RCVS were attributed to constriction of medium- and large-sized arteries, the extent of headache and vasoconstriction in RCVS was asynchronous [17–19]. This implicated that the headache might not be derived from medium- or large-sized arteries constriction. Ducros et al. demonstrated that headache could onset 1 week before vasoconstriction of large- and medium-sized arteries appeared, and in the same period the complications of cortical subarachnoid hemorrhage (cSAH), intracerebral hemorrhage (ICH), and reversible posterior leukoencephalopathy syndrome (RPLS) were observed. The ischemic events, including TIAs and cerebral infarction, often occurred after approximately 7 days. Based on the temporal pattern, their study illustrated that the underlying disturbance in the control of cerebral arterial tone first involved small distal arteries responsible for hemorrhages and RPLS and then progressed towards medium- and large-sized arteries responsible for ischemic events. Furthermore, the study also speculated that the headache of RCVS might primarily be due to the involvement of small distal arteries, with sudden changes in caliber (constriction or dilatation) that could stimulate perivascular pain-sensitive fibers [18, 20]. Thus, we inferred that myocardial ischemia might activate the sympathetic system, causing small intracranial arteries constriction and leading to a headache attack. Secondly, the cortical hypoperfusion might induce the occurrence of cortical spreading depression (CSD) in primary headaches, especially in a migraine [21]. Several experiments showed that CSD could contribute to the headache via activating meningeal nociceptors [22, 23] and activating or disinhibiting the second-order neurons in the trigeminocervical complex (TCC) [22, 24, 25]. Hence, according to the phenomenon of cerebral hypoperfusion, we proposed another possible mechanism: intracranial arteries constriction derived from myocardial ischemia lead to a CSD, which in turn caused a headache.

The field also proposed several other hypotheses to illustrate the mechanisms of CC. The first hypothesis was the convergence projection mechanism based on the fact that visceral afferent nerves from the heart and somatic afferent nerves from the head converged in the same spinal segments (C1 and C2 segments) belonging to TCC [26, 27]; when cardiac ischemic occurred, TCC was activated, leading to headache, similar to the reference pain of angina. The second hypothesis is hyper intracranial pressure mechanism: the sudden reduction of cardiac output associated with cardiac ischemia increased pressure in the left ventricle and in the right atrium, which might cause a decrease in venous return from the brain and subsequently an elevation of intracranial pressure, causing the headache [1, 4, 28, 29]. The third hypothesis is neurochemical mediator mechanism: during cardiac ischemia, several chemical mediators such as bradykinin, histamine, and substance P are released into the blood. These vasodilators could give rise to headache by dilation of the cerebrovasculature [1, 30]. However, these hypotheses were contrary to the fact that nitrates, also vasodilators, could relieve CC. Moreover, the PWI in the current case suggested microvessels constriction rather than vessel dilation during headache, which was also converse to this hypothesis.

### Conclusions

We presented a CC patient with cortical hypoperfusion during headache attack, which had never been reported. Based on the phenomenon, we proposed vessel constriction hypothesis including two possible physiological mechanisms to explicate the pathophysiology of CC. Firstly, when myocardial ischemia occurred, the sympathetic system was activated causing small intracranial arteries constriction and leading to a headache, similar to the mechanisms of RCVS. Secondly, hypoperfusion might induce CSD that might contribute to the headache confirmed by several experiments.

## Abbreviations

CC: Cardiac cephalalgia
cSAH: cortical subarachnoid hemorrhage
CSD: Cortical spreading depression
DWI: Diffusion-weighted imaging
ECG: Electrocardiogram
ICH: Intracerebral hemorrhage
LAD: Left anterior descending
LCX: Left circumflex
MRA: Magnetic resonance angiography
NSTEMI: Non-ST-elevation myocardial infarction
PTCA: Percutaneous transluminal coronary angiography
PWI: Perfusion-weighted images
RCA: Right coronary arteries
RCVS: Reversible cerebral vasoconstriction syndrome
RPLS: Reversible posterior leukoencephalopathy syndrome
TCC: Trigeminocervical complex

## References

[1] Grace A, Horgan J, Breathnach K (1997). Anginal headache and its basis. Cephalalgia.

[2] Headache Classification Committee of the International Head- ache Society (IHS) (2013). The international classification of headache disorders, 3rd edition (beta version). Cephalalgia.

[3] Lanza GA, Sciahbasi A, Sestito A, Maseri A (2000). Angina pectoris: a headache. Lancet.

[4] Yang Y, Jeong D, Jin DG (2010). A case of cardiac cephalalgia showing reversible coronary vasospasm on coronary angiogram. J Clin Neurol.

[5] Cutrer FM, Huerter K (2006). Exertional headache and coronary ischemia despite normal electrocardiographic stress testing. Headache.

[6] Amendo MT, Brown BA, Kossow LB (2001). Headache as the sole presentation of acute myocardial infarction in two elderly patients. Am J Geriatr Cardiol.

[7] Chen SP, Fuh JL, Wang SJ (2011). Reversible cerebral vasoconstriction syndrome: current and future perspectives. Expert Rev Neurother.

[8] Dodick DW (2002). Thunderclap headache. J Neurol Neurosurg Psychiatry.

[9] Gerretsen P, Kern RZ (2009). Reversible cerebral vasoconstriction syndrome: a hunderclap headache-associated condition. Curr Neurol Neurosci Rep.

[10] Edvardsson B, Persson S (2010). Reversible cerebral vasoconstriction syndrome associated with autonomic dysreflexia. J Headache Pain.

[11] Minisi AJ, Thames MD (1993). Distribution of left ventricular sympathetic afferents demonstrated by reflex responses to transmural myocardial ischemia and to intracoronary and epicardial bradykinin. Circulation.

[12] Webb SW, Adgey AA, Pantridge JF (1972). Autonomic disturbances at onset of acute myocardial infarction. Br Med J Clin Res.

[13] Randall WC, Hasson DM, Brady JV (1978). Acute cardiovascular consequences of anterior descending coronary artery occlusion in unanesthetized monkey. Proc Soc Exp Biol Med.

[14] Appel S, Kurityzi A, Zahavi I, Zigelman M (1992). Evidence for instability of the autonomic nervous system in patients with migraine. Headache.

[15] Schecter A, Stewart WF, Silberstein SD (2002). Migraine and autonomic nervous system function: a population based case control study. Neurology.

[16] Moulton EA, Becerra L, Johnson A (2014). Altered hypothalamic functional connectivity with autonomic circuits and the locus coeruleus in migraine. PLoS One.

[17] Chen SP, Fuh JL, Chang FC, Lirng JF, Shia BC, Wang SJ (2008). Transcranial color doppler study for reversible cerebral vasoconstriction syndromes. Ann Neurol.

[18] Ducros A, Fiedler U, Porcher R, Boukobza M, Stapf C, Bousser MG (2010). Hemorrhagic manifestations of reversible cerebral vasoconstriction syndrome: frequency, features, and risk factors. Stroke.

[19] Chen SP, Fuh JL, Wang SJ (2010). Magnetic resonance angiography in reversible cerebral vasoconstriction syndromes. Ann Neurol.

[20] Ducros A, Boukobza M, Porcher R, Sarov M, Valade D, Bousser MG (2007). The clinical and radiological spectrum of reversible cerebral vasoconstriction syndrome. A prospective series of 67 patients. Brain.

[21] Dalkara T, Nozari A, Moskowitz MA (2010). Migraine aura pathophysiology: the role of blood vessels and microembolisation. Lancet Neurol.

[22] Moskowitz MA, Nozaki K, Kraig RP (1993). Neocortical spreading depression provokes the expression of c-fos protein-like immunoreactivity within trigeminal nucleus caudalis via trigeminovascular mechanisms. J Neurosci.

[23] Zhang X, Levy D, Noseda R, Kainz V, Jakubowski M, Burstein R (2010). Activation of meningeal nociceptors by cortical spreading depression: implications for migraine with aura. J Neurosci.

[24] Zhang X, Levy D, Kainz V, Noseda R, Jakubowski M, Burstein R (2011). Activation of central trigeminovascular neurons by cortical spreading depression. Ann Neurol.

[25] Borysovych BV, Bogdanova OV, Lombard A (2015). Cortical spreading depression decreases Fos expression in rat periaqueductal gray matter. Neurosci Lett.

[26] Foreman RD, Garrett KM, Blair RW (2015). Mechanisms of cardiac pain. Compr Physiol.

[27] Akerman S, Romero-Reyes M (2013). Insights into the pharmacological targeting of the trigeminocervical complex in the context of treatments of migraine. Expert Rev Neurother.

[28] Wei JH, Wang HF (2008). Cardiac cephalalgia: case reports and review. Cephalalgia.

[29] Wang WW, Lin CS (2008). Headache angina. Am J Emerg Med.

[30] Kuo DC, Oravitz JJ, DeGroat WC (1984). Tracing of afferent and efferent pathways in the left inferior cardiac nerve of the cat using retrograde and transganglionic transport of horseradish peroxidase. Brain Res.

[31] Lipton RB, Lowenkopf T, Bajwa ZH (1997). Cardiac cephalgia: a treatable form of exertional headache. Neurology.

[32] Lance JW, Lambros J (1998). Unilateral exertional headache as a symptom of cardiac ischemia. Headache.

[33] Rambihar VS (2001). Headache angina. Lancet.

[34] Auer J, Berent R, Lassnig E, Eber B (2001). Headache as a manifestation of fatal myocardial infarction. Neurol Sci.

[35] Martínez HR, Rangel-Guerra RA, Cantú-Martínez L (2002). Cardiac headache: hemicranial cephalalgia as the sole manifestation of coronary ischemia. Headache.

[36] Famularo G, Polchi S, Tarroni P (2002). Headache as a presenting symptom of acute myocardial infarction. Headache.

[37] Gutiérrez-Morlote J, Pascual J (2002). Cardiac cephalgia is not necessarily an exertional headache: case report. Cephalalgia.

[38] Sathirapanya P (2004). Anginal cephalgia: a serious form of exertional headache. Cephalalgia.

[39] Chen SP, Fuh JL, Yu WC (2004). Cardiac cephalalgia. Case report and review of the literature with new ICHD-II criteria revisited. Eur Neurol.

[40] Korantzopoulos P, Karanikis P, Pappa E (2005). Acute non-ST-elevation myocardial infarction presented as occipital headache with impaired level of consciousness--a case report. Angiology.

[41] Gutierrez MJ, Fernandez JM, Timiraos JJ (2005). Cardiac cephalgia: an under diagnosed condition?. Rev Esp Cardiol.

[42] Seow VK, Chong CF, Wang TL (2007). Severe explosive headache: a sole presentation of acute myocardial infarction in a young man. Am J Emerg Med.

[43] Broner S, Lay C, Newman L (2007). Thunderclap headache as the presenting symptom of myocardial infarction. Headache.

[44] Dalzell JR, Jackson CE, Robertson KE (2009). A case of the heart ruling the head: acute myocardial infarction presenting with thunderclap headache. Resuscitation.

[45] Sendovski U, Rabkin Y, Goldshlak L (2009). Should acute myocardial infarction be considered in the differential diagnosis of headache?. Eur J Emerg Med.

[46] Chatzizisis YS, Saravakos P, Boufidou A (2010). Acute myocardial infarction manifested with headache. Open Cardiovasc Med J.

[47] Costopoulos C (2011). Acute coronary syndromes can be a headache. Emerg Med J.

[48] Elgharably Y, Iliescu C, Sdringola S (2013). Headache: a symptom of acute myocardial infarction. Eur J Cardiovasc Med.

[49] Asvestas D, Vlachos K, Salachas A (2014). Headache: an unusual presentation of acute myocardial infraction. World J Cardiol.

[50] Mathew PG, Boes CJ, Garza I (2015). A tale of Two systems: cardiac cephalalgia vs migrainous thoracalgia. Headache.

[51] Prakash S, Panchani N, Rathore C (2016). Cardiac cephalalgia: first case from India. Ann Indian Acad Neurol.

[52] Huang CC, Liao PC (2016). Heart attack causes headache - cardiac cephalalgia. Acta Cardiol Sin.

[53] Shankar A, Allan CL, Smyth D (2016). Cardiac cephalgia: a diagnostic headache. Intern Med J.

